# Development of a universal endogenous qPCR control for eukaryotic DNA samples

**DOI:** 10.1186/s13007-020-00597-2

**Published:** 2020-04-16

**Authors:** Cecilia Mittelberger, Lisa Obkircher, Vicky Oberkofler, Alan Ianeselli, Christine Kerschbamer, Andreas Gallmetzer, Yazmid Reyes-Dominguez, Thomas Letschka, Katrin Janik

**Affiliations:** 1 Applied Genomics and Molecular Biology, Institute for Plant Health, Laimburg Research Centre, Pfatten, Italy; 2 Virology and Diagnostics, Institute for Plant Health, Laimburg Research Centre, Pfatten, Italy

**Keywords:** Quantitative real-time PCR, Pathogen detection, Endogenous control, Reference gene, Phytoplasma

## Abstract

**Background:**

Phytoplasma are obligate intracellular plant-pathogenic bacteria that infect a broad range of plant species and are transmitted by different insect species. Quantitative real-time PCR (qPCR) is one of the most commonly used techniques for pathogen detection, especially for pathogens that cannot be cultivated outside their host like phytoplasma. PCR analysis requires the purification of total DNA from the sample and subsequent amplification of pathogen DNA with specific primers. The purified DNA contains mainly host DNA and only a marginal proportion is of phytoplasmal origin. Therefore, detection of phytoplasma DNA in a host DNA background must be sensitive, specific and reliable and is highly dependent on the quality and concentration of the purified DNA. DNA quality and concentration and the presence of PCR-inhibitors therefore have a direct impact on pathogen detection. Thus, it is indispensable for PCR-based diagnostic tests to validate the DNA preparation and DNA integrity before interpreting diagnostic results, especially in case that no pathogen DNA is detected. The use of an internal control allows to evaluate DNA integrity and the detection of PCR-inhibiting substances. Internal controls are generally host-specific or limited to a defined group of related species. A control suitable for the broad range of phytoplasma hosts comprising different insect and plant species is still missing.

**Results:**

We developed a primer and probe combination that allows amplification of a conserved stretch of the eukaryotic 28S rDNA gene. The developed endogenous qPCR control serves as a DNA quality control and allows the analysis of different eukaryotic host species, including plants, insects, fish, fungi, mammals and human with a single primer/probe set in single- or multiplex assays.

**Conclusions:**

Quality and performance control is indispensable for pathogen detection by qPCR. Several plant pathogens are transmitted by insects and have a broad range of host species. The newly developed endogenous control can be used with all so far tested eukaryotic species and since multiplexing is possible, the described primer and probe set can be easily combined with other PCR-based pathogen detection systems.

## Background

Polymerase chain reaction (PCR), first described by [1] and implemented as technique by [2], has become a broadly used technology in molecular biology in the last decades. The amplification of specific DNA in a given sample is a central technique important for many scientific and diagnostic methods and approaches [3]. The basic principle of PCR is the detection of DNA sequences specific for a certain organism, virus or an artificial DNA fragment. The DNA is amplified by using two oligonucleotides, so called primers, that specifically anneal to the flanking regions of the DNA of interest. In the presence of free nucleotides, a DNA polymerase then amplifies the stretch between the primers and the thereby exponentially amplified DNA (the amplicon) is stained with DNA intercalating dyes and can be detected or quantified by various methods [4].

The integrity of the target DNA, i.e. the template is crucial for a successful PCR reaction [5]. However, in case the template DNA was (i) lost or (ii) degraded during the sample preparation procedure or (iii) amplification inhibiting substances are present in the sample, the PCR might fail although the DNA of interest was present in the sample. Such failures lead to false-negative results and must be avoided to provide highly reliable PCR results. To analyze the unwanted loss of DNA during a purification procedure exogenous DNA of known sequence can be added to a sample prior to DNA extraction [6]. This specific exogenous DNA must then be detected by PCR, as an internal amplification control in sufficient amounts after extraction to ensure that DNA was successfully purified from the sample. The use of exogenous controls also allows to analyze the presence of potential PCR inhibitors in the sample. Nonetheless, this method is based on the addition of an external DNA fragment and does not deliver information about the integrity of the target DNA that is of real interest [7].

Beside exogenous PCR controls, endogenous controls can be used. An endogenous PCR control provides information about the successful purification of DNA from the actual sample and the absence of inhibiting substances in the extracted DNA [8]. Endogenous thus means that the control DNA is present in the sample but is not the target i.e. host DNA during pathogen detection or reference genes (or RNA/cDNA, respectively) in expression studies [9]. Successful amplification of the endogenous control allows to deduce that the DNA (i) was successfully purified from the sample, (ii) was not degraded during the procedure and that (iii) the PCR sample does not contain certain substances in a quantity that hampers successful DNA amplification. Furthermore, the amplification of a target of known quantity (e.g. the number of copies per genome or a housekeeping gene that is invariantly expressed) can be utilized as a reference for quantification of the target DNA [10]. So far, no universal endogenous control has been described that can be used for phylogenetically highly diverse samples.

Phytoplasma infect several hundred plant species and cause severe damages in a wide range of crop plant species [11, 12]. They are transmitted by different insect vectors [11] and thus phytoplasma surveys often involve the detection of these bacteria in different plant and insect species. Nowadays, most tools for phytoplasma detection are based on quantitative PCR (qPCR) techniques, using intercalating DNA dyes (SYBR-Green) or hybridization probes (TaqMan^®^) [13]. Several qPCR protocols are available for the detection of phytoplasmas using different host specific endogenous internal controls [13–19]. Some of the available host specific internal controls can be used simultaneously with the phytoplasma specific primers in a multiplex qPCR assay [14, 15, 18, 19], for others it is necessary to perform a separate qPCR run [20]. However, a universal endogenous control for the simultaneous detection of phytoplasma specific targets and phylogenetically different host species DNA is still missing.

The aim of this work was the development of an endogenous control that can be used as an indicator of DNA purification and integrity for several different eukaryotic host species. This control was sought to be combined with the PCR-based detection of phytoplasma to establish a reliable detection method in different (potential) hosts using TaqMan^®^ chemistry.

In this study a method is described that comprises the amplification of a conserved, short stretch of the 28S rDNA gene as a universal endogenous control. Beside the primers a hydrolysis probe was designed containing locked nucleic acids (LNA) to design a short probe with high temperature melting properties and improved binding strength even in single mismatch nucleotides [21, 22]. The combination of probe and primers allows fluorometric single- or multiplex amplicon detection using TaqMan^®^ qPCR assays.

## Results

The objective of this study was the development of a qPCR assay that incorporates the amplification of an endogenous control gene to the pathogen detection in different host DNA samples. Thus, a highly conserved region that might be suitable for the development of universal primers and probe among phylogenetic distinct species was identified within the 28Sr DNA stretch. The newly developed primer and probe combination was first optimized for qPCR application using SYBR-Green and TaqMan^®^ chemistry and then tested in different assays to validate its performance.

### Validation of qPCR performance

**Fig. 1:**
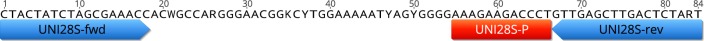
Consensus sequence of conserved 28S rDNA stretch. Consensus of sequence alignment of the conserved region of 28S V. The sequence is depicted from 5′ (left) to 3′ (right). Primer and probe binding sites are indicated by blue (primers) and red (probe) bars

The assay validation was performed following the MIQE [7] guidelines (see Additional file 1: Table S1). The primers UNI28S-fwd and UNI28S-rev (Fig. 1) were used in a SYBR Green qPCR assay to determine the optimal reaction and cycling conditions.

**Fig. 2:**
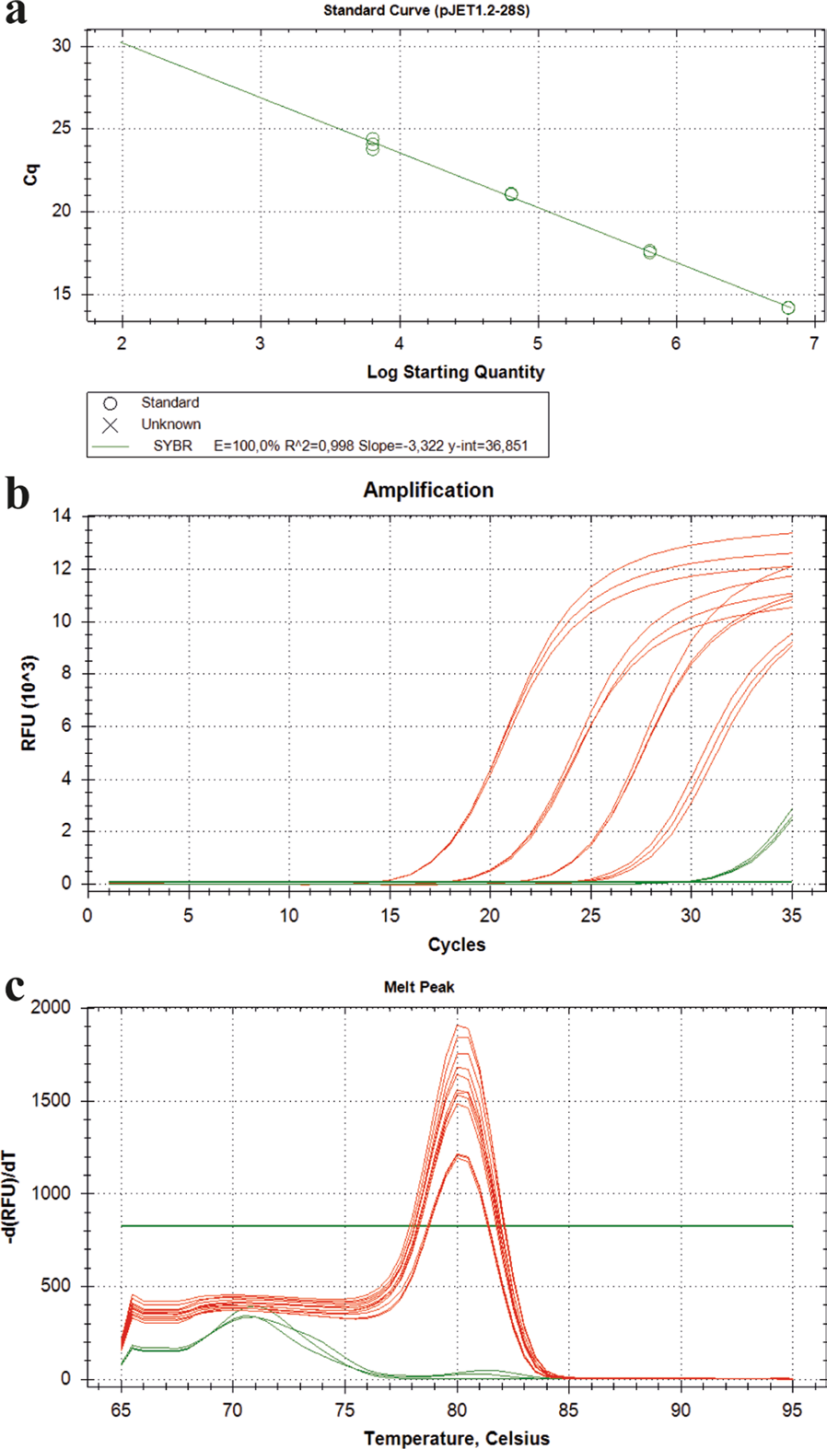
Amplification with UNI28S primers in a SYBR-Green assay. Amplification of 28S using the UNI28 primers in a SYBR-Green assay. A serial dilution of pJET1.2-28S was prepared covering tenfold dilutions from 6.38 × 10^6^ to 6.38 × 10^3^ copies per reaction. Each dilution step was run as a triplicate. Red lines depict the samples containing the plasmid template in different dilutions and the green line represents the no template control (NTC; nuclease free water instead of template)

Using the primers at annealing temperatures from 50 to 60 °C uniform amplicons could be generated (see Additional file 1: Table S2). Specific melting curve peaks were observed in all samples (see Additional file 1: Fig. S1) and the broad annealing temperature range indicates that the amplification is robust [23]. To determine the optimal primer concentrations for the assay (i.e. concentrations that allow reliable amplification in the absence of primer dimer formation) a primer concentration matrix was prepared. This analysis revealed that a primer concentration of 250 nM forward and 250 nM of the reverse primer in the final reaction allowed amplification of a specific amplicon as characterized by a low C_q_ value and specific melting curves and did not lead to primer dimer formation (see Additional file 1: Table S3 and Fig. S2). Higher primer concentrations increased the fluorescent signal during amplification (characterized by a lower C_q_) but led to primer dimer formation. The addition of MgCl_2_ to the PCR mix did not improve, but impaired amplification as characterized by an increasing C_q_ value (see Additional file 1: Fig. S3). Serial dilutions of pJET1.2-28S revealed that the linear dynamic range (LDR) in which a PCR efficiency between 95 and 105% and an R^2^ of 0.99 could be achieved, was between 6.38 × 10^3^ and 6.38 × 10^6^ copies per reaction in the SYBR-Green based qPCR assay (Fig. 2).

For optimization of probe-based qPCR, primer performance was analyzed in combination with the described hydrolysis probe. Since primer dimers are not interfering with the amplicon detection, the primer concentration was increased to 400 nM of each primer in order to enhance the amplification reaction (see Additional file 1: Fig. S2).

**Fig. 3:**
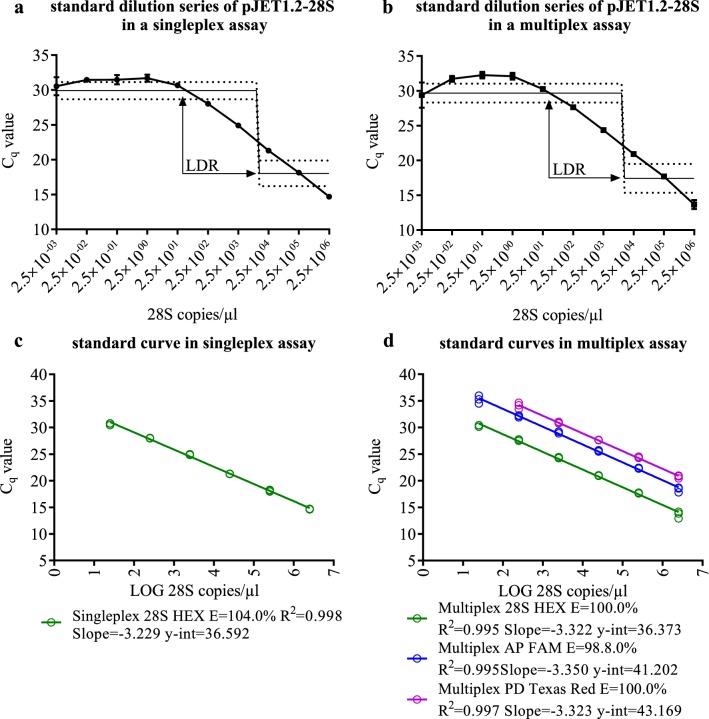
Standard dilution series of plasmid standards. 10 point tenfold plasmid standard dilution series with **a** only 28S plasmid standard (singleplex) or **b** a combination of three different plasmid standards (multiplex) run in triplicates using a TaqMan^®^ assay and standard curves of the 28S plasmid and plasmid standards with phytoplasmal target including performance parameters of **c** singleplex and **d** multiplex assay The linear dynamic range is depicted as interpolation line with 95% confidence interval (dotted line) and calculated by a 4-point logarithmic nonlinear regression analysis. Error bars represent the SEM of the three technical triplicates

The LDR as well as the limit of detection (LOD) were determined by using a tenfold standard dilution series of pJET1.2-28S between 5 × 10^6^ and 5 × 10^−3^ copies per reaction. Dilutions containing the theoretical amount of less than one copy per reaction served to reach the C_q_ plateau in which no amplification occurs or in which the plasmid quantity cannot be linearly correlated to the obtained C_q_. The LDR could be reliably detected and ranged between 54.24 (C_q_ = 29.91) and 287,818 (C_q_ = 18.04) plasmid copies per reaction (Fig. 3a).

To determine if the UNI28S primers and probe can be used for multiplexing, an exemplary multiplex TaqMan^®^ qPCR assay was performed to detect two economically relevant phytoplasma species: apple proliferation (AP) phytoplasma (‘*Candidatus* Phytoplasma mali’) and pear decline (PD) phytoplasma (‘*Candidatus* Phytoplasma pyri’) together with the eukaryotic 28S rDNA in different concentrations. The LDR, with a range of 64.51 (C_q_ = 29.67) to 447,235 (C_q_ = 17.43) plasmid copies per reaction, for the detection of the 28S rDNA in a multiplex assay, was found to be comparable to the singleplex assay (Fig. 3a, b). The performance of the 28S amplification was 100% ± 5% (max) and an R^2^ > 0.99 in the singleplex and the multiplex assay (Fig. 3c, d).

The LOD was found to be five 28S copies per reaction (2.5 copies/µl), since this was the lowest number of 28S copies that could reliably detected in repeated assays using the pJET1.2-28S calibration curve.

**Fig. 4:**
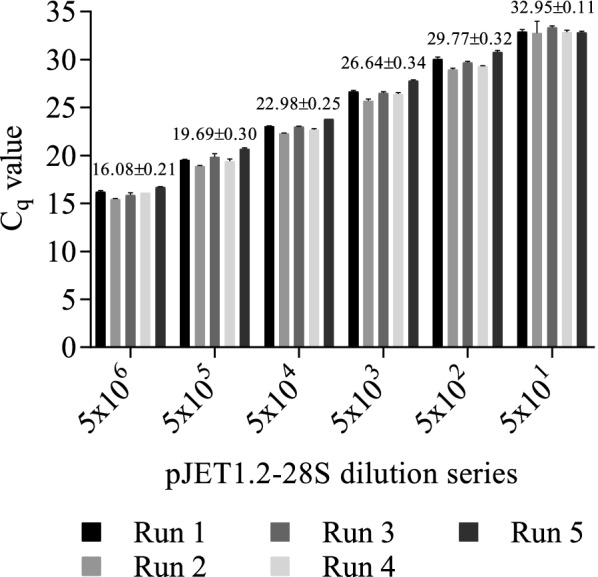
Intraassay and interassay variation. Intraassay and interassay variation of five independent TaqMan^®^ qPCR assays. A six-point serial dilution series of pJET1.2-28S was used as the template for amplification. The bars represent the mean C_q_-value of three technical replicates ± SEM (error bars, intraassay variation) and the values over the grouped columns show the mean C_q_ value ± SEM of the five independent runs (interassay variation). Differences were analyzed using Two-way ANOVA with Tukey’s multiple comparison posttest

For determination of the repeatability each sample was run in triplicates and the standard deviation of the measurement was calculated. Intraassay variation was determined for five independent runs using four different template concentrations (Fig. 4). The average C_q_-value variation of the technical triplicates was maximum 5.1% (based on the average C_q_-value) within the same run (Fig. 4). Based on the comparison of five independent runs, using the same four-point standard curve measured in triplicates, that had a similar intercept (38.74 ± 0.35), the average C_q_-value of each individual standard dilution did not vary more than 3.7% (based on the average C_q_-value of all five independent runs) between the assays (Fig. 4). PCR amplification efficiency ranged between 95.8 and 101.7%, with an R^2^ ≥ 0.99.

The primers were designed to amplify a 28S rDNA fragment of eukaryotic organisms. Specificity was therefore determined with DNA from prokaryotic *E. coli* as a negative control. Primer and probe combination in assays with *E. coli* DNA as the template did not generate an amplicon under the described PCR conditions (Table 1). The primer and probe combination amplified the 28S rDNA fragment in all 43 tested eukaryotic species (Table 1) and in cDNA samples from *Malus* × *domestica*, *Vitis vinifera* and *Cacopsylla picta*.

**Table 1:** Amplification results of eukaryotic species

Taxon	Species	Tested tissue	Mean C_q_	Stdv C_q_	28 S copies reaction^−1^
Arthropod	*Anaceratagallia ribauti*	Whole insect	16.40	0.17	2,605,892
Arthropod	*Aphrophora alni*	Whole insect	8.65	0.72	561,670,136
Arthropod	*Asymmetrasca decedens*	Whole insect	16.85	0.27	1,916,323
Arthropod	*Cacopsylla melanoneura*	whole insect	16.52	0.02	14,145,566
Arthropod	*Cacopsylla picta*	Whole insect	16.24	0.07	17,121,902
Arthropod	*Cicadula quadrinotata*	Whole insect	14.51	0.08	9,706,053
Arthropod	*Cixius nervosus*	Whole insect	10.22	0.72	189,127,348
Arthropod	*Dicranotropis hamata*	Whole insect	13.84	0.19	15,409,055
Arthropod	*Edwardsiana rosae*	Whole insect	15.18	0.04	6,085,580
Arthropod	*Emelyanoviana mollicula*	Whole insect	16.18	0.14	3,042,300
Arthropod	*Empoasca vitis*	Whole insect	19.31	0.16	348,147
Arthropod	*Eriosoma lanigerum*	Whole insect	15.91	0.18	119,623,912
Arthropod	*Ixodida*^a^	Whole insect	21.54	0.65	133,850
Arthropod	*Laodelphax striatella*	Whole insect	12.81	0.23	31,543,702
Arthropod	*Macrosteles quadripunctulatus*	Whole insect	20.38	0.12	165,800
Arthropod	*Macrosteles cristatus*	Whole insect	16.53	0.16	2,381,296
Arthropod	*Macrosteles laevis*	Whole insect	16.02	0.11	3,407,075
Arthropod	*Macrosteles ossiannilssoni*	Whole insect	15.15	0.06	6,213,481
Arthropod	*Macrosteles sexnotatus*	Whole insect	17.33	0.07	1,373,854
Arthropod	*Psammotettix alienus*	Whole insect	16.20	0.09	3,007,348
Arthropod	*Psammotettix confinis*	Whole insect	15.06	0.07	6,598,274
Arthropod	*Stictocephala bisonia*	Whole insect	9.42	0.90	330,858,020
Arthropod	*Zygina flammigera*	Whole insect	16.78	0.17	2,011,619
Arthropod	*Zyginidia pullula*	Whole insect	15.88	0.38	3,745,691
Fish	*Salmo salar*^a^	Meat	20.69	0.11	240,091
Fungi	*Boletus edulis*^a^	Stem part	15.42	0.28	9,270,902
Fungi	*Saccharomyces cerevisiae*^a^	Colony	17.97	0.25	1,584,262
Mammal	*Bos primigenius taurus*^a^	Meat	25.08	0.31	11,466
Mammal	*Capreolus capreolus*^a^	Meat	22.57	0.57	65,416
Mammal	*Equus ferus caballus*^a^	Hair	22.95	0.39	50,274
Mammal	*Homo sapiens*^a^	Blood	26.57	0.93	4,103
Mammal	*Mus musculus*^a^	Blood	24.09	0.23	22,820
Mammal	*Ovis gmelini aries*^a^	Meat	22.19	0.36	84,923
Mammal	*Sus scrofa*^a^	Meat	23.66	1.05	30,811
Plant	*Ginkgo biloba*^a^	Leaf	19.89	0.23	418,889
Plant	*Lycopersicon esculentum*^a^	Leaf	15.40	0.05	8,605,181
Plant	*Malus* × *domestica*^a^	Root	20.73	1.69	233,529
Plant	*Nicotiana occidentalis*^a^	Leaf	16.95	0.15	2,989,473
Plant	*Olea europaea*^a^	Leaf	20.28	1.18	319,704
Plant	*Pinus cembra*^a^	Leaf	17.68	0.31	1,932,346
Plant	*Prunus armeniaca*^a^	Leaf	16.25	0.25	5,216,449
Plant	*Pyrus communis*^a^	Leaf	20.72	0.53	235,152
Plant	*Vitis vinifera*^a^	Leaf	18.04	0.21	1,509,260

Amplification results of the 28S rDNA amplicon in 43 different eukaryotic species with threshold cycle values (C_q_) and quantification results based on the tenfold standard dilution series of pJet1.2-28S

^a^Template DNA was diluted to 5 ng/µl

The quantification of the 28S amplicon in 23 different species, using the same mass of template DNA showed huge differences between the tested species (Table 1). While in 1 ng of *Boletus edulis* DNA more than 900,000 copies of the 28S fragment were detected, in the same quantity of a human blood DNA sample only 410 copies were amplified.

### Comparison with different other internal controls

**Fig. 5:**
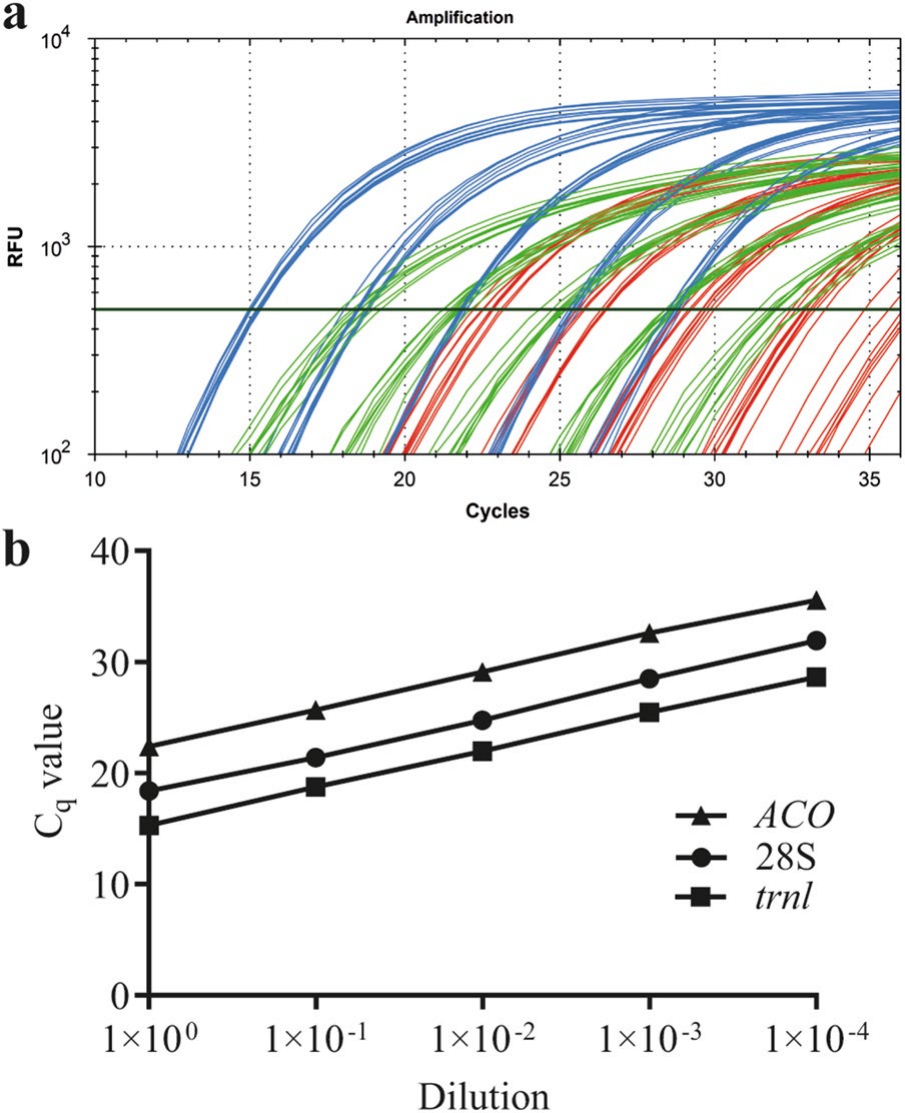
Comparison between different endogenous controls. Amplification curves of 28S (green), *cpLeu* (blue) and *ACO* (red) reference genes of a tenfold dilution series (undiluted—1:10,000) from three different apple root samples, measured as triplicates (**a**) and the five point tenfold dilution series of sample 1 (**b**) measured in triplicates with the three reference genes

To validate whether the performance of the qPCR using the UNI28S primers and probe is comparable to the performance of internal controls that are currently used in phytoplasma detection assays, exemplarily the amplification performance of two different apple specific gene fragments was compared to the performance of the qPCR with 28S rDNA as the target. An apple specific chloroplast DNA gene fragment of the tRNA leucine (*trnL*) gene [24] and a fragment of the single-copy gene 1-aminocyclopropane-1-carboxylate oxidase (*ACO*) [25] were used for this analysis. While a reliable detection of *ACO* in highly diluted apple DNA templates (1:10,000) was difficult and only reliable in up to 1:1000 diluted samples, 28S and *cpLeu* reliably amplified even 1:10,000 diluted samples (see Additional file 1: Table S4) and showed thus a lower LOD than *ACO* (Fig. 5).

### TaqMan^®^ assay vs SYBR-Green assay

**Fig. 6:**
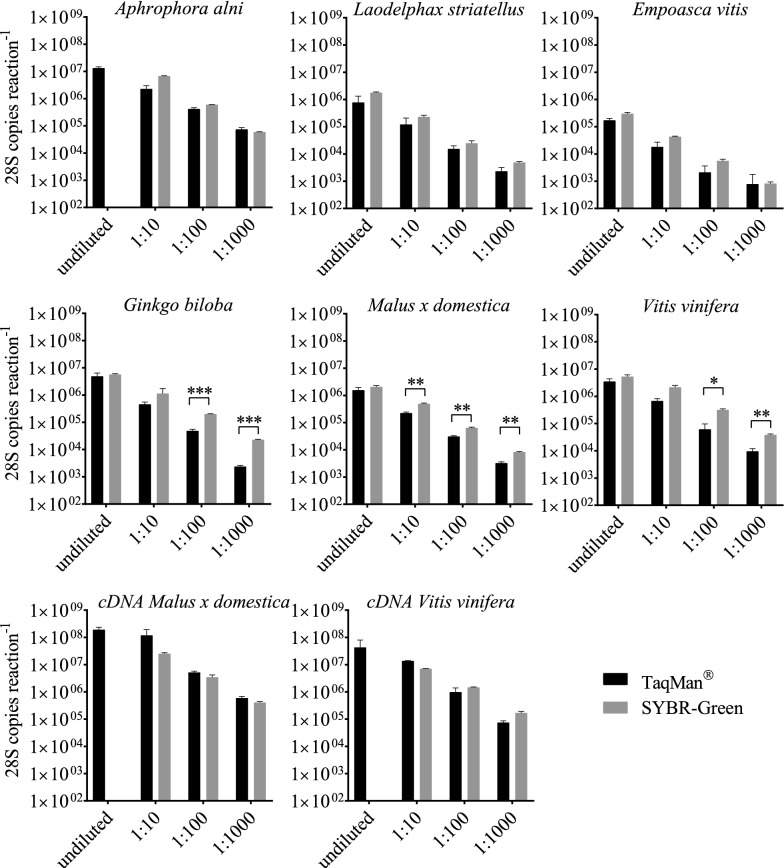
Comparison between SYBR-Green and TaqMan^®^ chemistry. Amplification of the 28S conserved fragment in four-point tenfold sample dilutions of *Aphrophora alni*, *Laodelphax striatellus*, *Empoasca vitis*, *Ginkgo biloba*, *Malus* × *domestica*, *Vitis vinifera* and cDNA samples of *Malus* × *domestica* and *Vitis vinifera* run as triplicates. Two different amplification chemistries were used, i.e. SYBR-Green and TaqMan^®^. Error bars represent SEM and significant differences were determined by multiple t-test with Holm-Sidak correction indicated by *P ≤ 0.05, **P ≤ 0.01, ***P ≤ 0.001

Four-point tenfold dilution series from different samples (*A. alni, L. stri, E. vitis, Ginkgo biloba, Malus* × *domestica, Vitis vinifera*, *Malus* × *domestica* cDNA, *Vitis vinifera* cDNA), tested in triplicates, were used as templates for the detection of the 28S fragment in a TaqMan^®^ and SYBR-Green assay (Fig. 6). The quantification results from the two assays are comparable. Nevertheless, when dealing with undiluted samples with a high DNA amount, it was not always possible to get reliable results when using SYBR-Green (see Fig. 6, *A. alni*, *Malus* × *domestica* cDNA, *Vitis vinifera* cDNA) and quantification results from five diluted samples differed significantly between the two assays.

### Limitations of the SYBR-Green assay

**Fig. 7:**
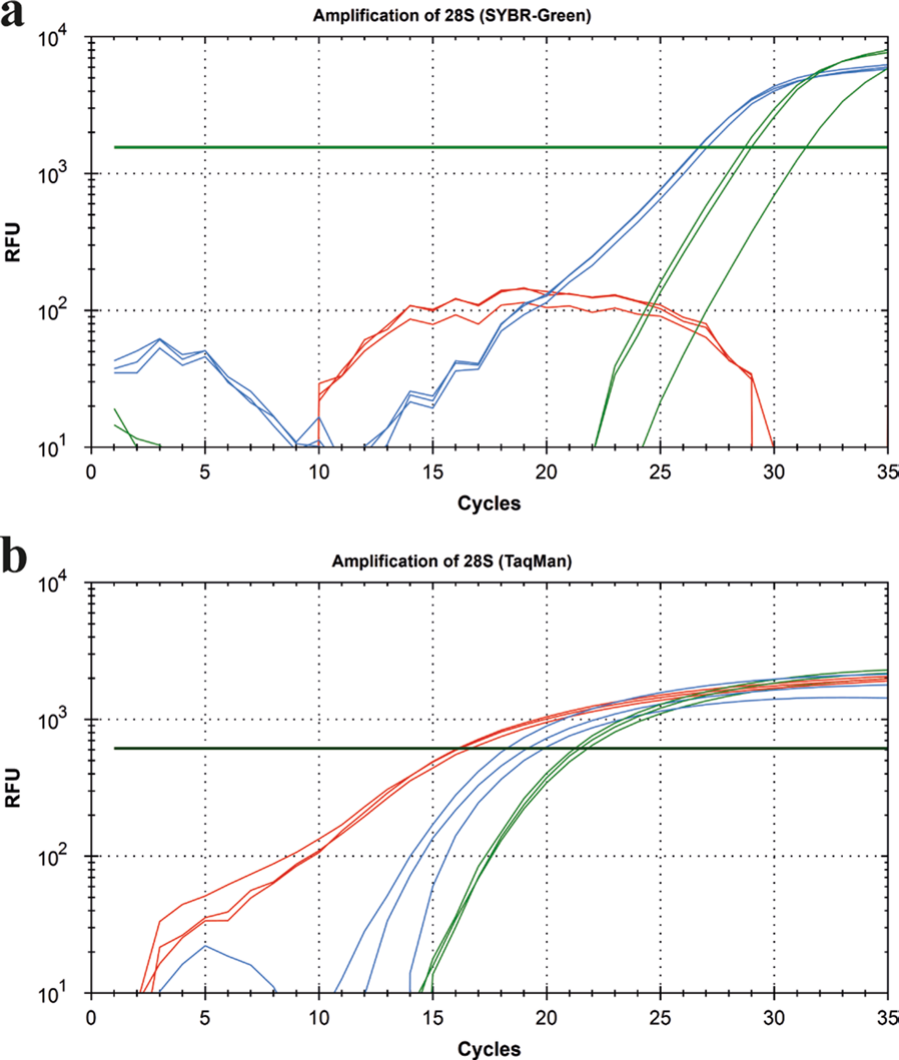
Limitations of SYBR-Green assay. Amplification curves of 28S fragment in an undiluted (red), 1:10 (blue) and 1:100 (green) diluted *Aphrophora alni* sample using a SYBR-Green (**a**) and TaqMan^®^ (**b**) assay. Each dilution step was run as a triplicate. The undiluted sample did not show reliable amplification curves when using SYBR-Green

Even though primer concentrations were optimized for SYBR-Green based qPCR assays, reactions with a high template DNA amount showed problems during amplification (Fig. 7). It was necessary to dilute those samples up to 100-fold to gain reliable results when using SYBR-Green for 28S amplification. Those problems appeared especially with big insect species, e.g. *Aphrophora alni*, where DNA was extracted from the whole insect body and thus the extracted DNA amount was relatively high (182.5 ± 1.4 ng µl^−1^) compared to smaller insect species (e.g. *Empoasca vitis*, 4.4 ± 0.6 ng µl^−1^) or DNA from 100 mg of plant tissue (e.g. *Vitis vinifera*, 23.9 ± 0.1 ng µl^−1^).

In contrast to the SYBR-Green assay, TaqMan^®^ assays worked reliably even in samples with a high DNA background.

## Discussion

This study describes a new primer and probe combination that allows amplification of a conserved stretch of the eukaryotic 28S rDNA gene. The amplification of this gene fragment can be used for sensitive detection of eukaryotic DNA, coming from phylogenetic highly distinct species, for different purposes. The use of internal controls in qPCR applications is particularly important to verify DNA integrity and the absence of PCR inhibitors from the sample. PCR-based detection of pathogen specific DNA in a background of eukaryotic DNA is a common technique for diagnostic purposes. Pathogen detection can fail in a given sample due to the absence of the pathogen in the sample or due to impairments during DNA preparation or PCR performance. These impairments can be of different origin, such as due to low quality DNA extraction, DNA degradation, presence of inhibitors in the DNA sample or a high DNA background to only mention a few. Factors leading to PCR impairment can hamper pathogen detection or lead to false-negative results. It is therefore indispensable to ensure that DNA has been successfully extracted from the respective tissue and that PCR inhibitors are absent in the DNA. Such a quality control can be achieved by amplifying a known DNA target that has been added to the sample prior to DNA extraction (exogenous control; exCtrl) or by amplifying a DNA target present in the extracted sample DNA (endogenous control; enCtrl). Successful amplification of the enCtrl indicates that intact DNA has been successfully extracted and that PCR inhibition does not occur. Furthermore, it allows relative, non-spectrometric DNA quantification. EnCtrls are thus preferable over exCtrls because they allow verification of DNA integrity and prove the absence of PCR inhibitors [9]. Moreover, enCtrls can be used for the relative quantification of pathogens in a given sample [13].

When working with phytoplasma, it is important to have reference genes for a broad range of plant or insect host species available. Although several protocols are available for the detection of different phytoplasma species in single- or multiplex assays [14, 16–18, 24], all of them use internal endogenous controls with a rather narrow host range. Only the 18S rDNA gene has been reported as a universal endogenous qPCR control, working with a broad range of plant species [26]. However, a universal enCtrl that amplifies a conserved DNA target present in diverse eukaryotic species including plants and insects has not been reported so far.

For reverse transcription (RT)-qPCR applications several reference genes are available as enCtrl. However, available sequences for primer and probes of different reference genes are not suitable for a universal detection among phylogenetic distinct species. In most cases sequences and assays are species specific, restricted to certain taxa or optimized for particular gene expression studies [27, 28]. Among the (RT)-qPCR enCtrl the 28S rDNA gene was found to be stably expressed under different experimental conditions [29], however the use as internal control in gene expression studies is controversially discussed [30]. The 28S rDNA gene is nuclear-encoded but not a single copy gene. Therefore, absolute quantification of genome copies in a sample is not possible if the number of 28S rDNA gene copies in the respective genome is unknown. But, since the 28S rDNA gene is nuclear-encoded it is possible to relatively quantify host DNA between different samples deriving from the same species. This would not be possible amplifying a target gene that is organelle-encoded (e.g. in the mitochondrial or chloroplast genome) since the number of organelles can be tissue-dependent. The comparison between the amplification curves of *trnL*, *ACO* and 28S illustrates the difference regarding the difference between a nuclear-encoded single-copy (*ACO*), a nuclear-encoded multi-copy (28S) and an organelle-encoded (*trnL*) gene. *TrnL* is encoded in the chloroplast, an organelle that occurs in high (but varying) numbers in each cell. One copy of *ACO* represents a single cell and the *28S* is nuclear encoded, but multi-copy, which means that the 28S copy-number does not vary between the cells of a given species but the absolute number of this gene per cell is unknown or needs to be determined. The amplification of the 28S target in a sample therefore generates lower C_q_s than the amplification of *ACO* but higher C_q_s compared to *trnL* as seen in Fig. 5. Due to its universality, the newly developed 28S-based enCtrl can be used in high throughput qPCR applications where several different species shall be analyzed regarding the presence of phytoplasma e.g. when searching for potential insect vectors. The host DNA quantity, as a proxy for DNA purification efficacy can be compared between samples from the same species but not between DNA samples derived from different species. In case that it is necessary to determine the 28S rDNA gene copy number present in the genome of a certain eukaryotic species, Southern Blot analysis can be performed. However, the determination of 28S rDNA gene copy number per genome or cell was not part of this study. Quantification of DNA using the described method is limited to eukaryotic DNA, that means that DNA of prokaryotic origin present in the sample is not detected. For the detection of phytoplasma this is of minor relevance since the quantity of total prokaryotic DNA in the sample is very low and thus neglectable. However, quantification of the 28S rDNA gene in a mixture of eukaryotic (host) and prokaryotic (phytoplasma) DNA is therefore rather an estimation than an exact quantification of total DNA present in the sample. Accurate quantification is achieved only for the eukaryotic DNA proportion in the sample. Beside the application of the newly developed universal enCtrl during phytoplasma detection, it can be used in combination with other qPCR assays whenever an enCtrl for a eukaryotic host species is necessary. Since it is also applicable for amplification of cDNA targets it might be also applicable for certain gene expression studies, however the suitability of the 28S rDNA gene as an accurate housekeeping gene should be carefully assessed [30–33].

A careful evaluation of qPCR results is crucial, because many factors can influence PCR performance and thus the accuracy of the results [34, 35]. It is recommended to analyze standards, i.e. serial dilutions of a sample containing the target DNA, in parallel in every assay [7, 36, 37]. As shown in Fig. 6 absolute quantification is depending on the chemistry applied in the qPCR. Even though the primers are the same in both assays and the PCR performance for both assay types was in the range of E = 100% ± 5% and R^2^ ≥ 0.99 the SYBR results showed significantly higher absolute quantities than the TaqMan^®^ assay at some dilutions. This might be explained by the fact that SYBR intercalates with all double stranded DNA present in the sample and does not stain the specific amplicon only. This might cause non-specific fluorescence especially in templates that contain a high amount of background DNA or in templates in which primer dimer formation occurs. Latter can lead to “false-high” results when using diluted DNA samples. These technical restrictions can lead to a narrow LDR when using SYBR. The LOD is not necessarily affected since the melting curve allows discrimination between the specific amplicon and primer dimers. However, a differential quantification between the fluorescence derived from the specific and the non-specific signal is impossible. In samples with high DNA background, even if the sample is free from inhibitors, SYBR can create false-negative results due to its unspecific DNA-binding characteristics. Detection in samples with high DNA concentrations is thus very likely to fail in any qPCR analysis that is based on intercalating DNA dyes. In TaqMan^®^ assays only the specific amplicon creates fluorescence. Background DNA and primer dimer thus do not lead to a non-specific fluorescence in this assay.

We used primer and probes described for the detection of two different phytoplasma species [42] in combination with the UNI28S primer and probe to evaluate the performance of the latter in a three-fluorophore multiplex assay. Performance validation of the UNI28S primers and probe combination was done according to the MIQE guidelines [7] with a plasmid standard dilution curve. The use of standard dilution curves renders it possible to measure and evaluate qPCR performance parameters, such as PCR efficiency. The semilogarithmic standard dilution curve should display a linear range with a low variation in the single points, cover the whole range of quantified DNA amount and display a high correlation coefficient and a slope near − 3.33, which is the slope of the LDR from the semilogarithmic transformation of the copies per reaction (y) and the measured C_q_ (x) in which an increment of one C_q_ corresponds to the doubling of DNA present in the sample (Fig. 3). A slope of this value is thus a numeric indicator that doubling of DNA in each cycle occurred and thus stands for 100% amplification or PCR efficiency (E) [38].

Outside the LDR, detection might be still possible, but the absolute quantity cannot be determined with the linear regression correlation between C_q_ and template concentration in the LDR. Minimum and maximum, i.e. the start and end point of the LDR, can be determined by 4-point logarithmic nonlinear regression analysis as depicted in Fig. 3a, b. The LOD, which is the lowest concentration that can be detected with reasonable certainty [7] is an indicator of PCR sensitivity. In our analytical assays an LOD of five template copies per reaction could be reliably detected. This number is almost equal to the most sensitive theoretically possible LOD of three copies per reaction [7]. However, it needs to be mentioned that the LOD is a value that needs to be determined in every novel PCR setting, especially when using different master mixes, cyclers, etc.

The LOD as numeric value for PCR sensitivity is an important and characteristic parameter. However, it was only of secondary relevance in our practical settings, since the quantity of 28S was not limiting. For the detection of low quantities of a target, i.e. for pathogen diagnostics the pathogen specific LOD is crucial and should be determined in a negative control DNA background of host DNA to mimic the actual assay situation. While the LOD is important for diagnostic approaches to detect plant pathogens, it is less important for reference gene detection. Nevertheless, it must be considered that if the host DNA amount is already very low, the probability to detect pathogen DNA in that respective sample is even lower. Comparison of the total DNA amount between samples is thus important to guarantee a certain diagnostic standard. In our experimental setting an accurate quantification of 28S rDNA copies and the determination of the LDR (but not the LOD) was therefore rather important to allow the comparison of DNA quantities from samples analyzed in independent assays. While the detection limit did not play a major role, high concentrations of DNA hampered SYBR-Green based detection of the target DNA. This is most probably because of the limited availability of DNA intercalating SYBR-Green dye in the reaction. The presence of high amounts of double stranded template DNA before the amplification can lead to a saturating incorporation of SYBR-Green dye. This would lead to a depletion of SYBR-Green available for the intercalation into newly formed amplicons during PCR amplification and thus prevent the formation of an increasing fluorescent signal during the reaction. The LOD of the 28S amplification using SYBR-Green was about 1000-fold higher compared to the LOD in the TaqMan^®^ assay.

The LDR of the SYBR-Green assay was determined between 6.38 × 10^3^ and 6.38 × 10^6^ and is thus also suitable for quantification in a decent range. However, with the limitation that very high DNA concentrations significantly hampered the amplification. Our results clearly indicate that the assay type strongly impacts the confidence interval in which target quantification can be achieved. Even if the same primers are used and these primers amplify in a broad melting temperature range, the master mix impacts the PCR performance as indicated by our SYBR-Green results with varying MgCl_2_ concentrations. These findings underline the importance to carefully evaluate and improve every PCR assay with appropriate standards and clearly define its characteristics in every new setting and choose the appropriate assay for the respective scientific question. It might be even more important to know the limitations for every respective assay to be able to interpret results. Although different studies report a comparable sensitivity of TaqMan^®^ and SYBR-Green based qPCR [39, 40] this is not a general rule, since it strongly depends on how neat the respective PCR assay has been established and under what conditions the assays are performed.

The described UNI28S primers and probe were successfully used in a multiplexing approach and are routinely used in our lab as an enCtrl during phytoplasma detection in different plant and insect species. Since the novel primers and hydrolysis-probe amplify a conserved fragment of the 28S rDNA gene in many diverse eukaryotic species, they can thus be used for many different research purposes.

## Conclusion

A universal endogenous control for phylogenetic distinct eukaryotic template DNA is now available for qPCR assays. Performance and quality control of qPCR runs is crucial for reliable results. EnCtrls in qPCR assays proof the integrity of template DNA and the absence of PCR inhibitors. Together with plasmid standards enCtrls can be useful for quantification purpose. The newly described and validated primer and probe combination is suitable as endogenous control in a broad range of eukaryotic species, and the single- as well as multiplex protocol can be used for pathogen detection based on qPCR assays.

## Methods

### Primer and probe design

To identify a highly conserved, short stretch in the eukaryotic genome, fragment V of the nuclear-encoded 28S rDNA was amplified with the respective forward and reverse primers described in [41]. The amplicons of approximately 760 bp were sequenced and aligned using Geneious version 11.1.4. (https://www.geneious.com). A conserved sequence stretch was identified, and suitable qPCR primers and a probe were designed that allow amplification of an 84 bp region (Fig. 1) generating an amplicon within the size range recommended for SYBR and probe-based assays [23]. To create a primer pair that universally amplifies in eukaryotic species, a wobble nucleotide was incorporated in the reverse primer UNI28S-rev, while for the probe development LNAs were integrated to increase the binding specificity and strength. The designed sequences for primer pair UNI28S-fwd/UNI28S-rev and the probe UNI28S-P are depicted in Table 2.

**Table 2:** Primer and probes of endogenous reference genes used in this study

Target gene	Target species	Primer name	5′–3′ sequence	References
28S	Eukaryotes	UNI28S-fwd	CTACTATCTAGCGAAACC	This work
		UNI28S-rev	AYTAGAGTCAAGCTCAAC	
		UNI28S-P	HEX-AAA + G+A + AG + A+C + C+C + T-DAB	
*cpLeu*	*Malus* × *domestica*	qMD_cpLeu-fwd	CCTTCATCCTTTCTGAAGTTTCG	[24]
		qMD_cpLeu-rev	AACAAATGGAGTTGGCTGCAT	
		qMD_cpLeu	HEX-TGGAAGGATTCCTTTACTAAC-TAMRA	
*aco*	*Malus* × *domestica*	Md-ACO-F	CCAGAATGTCGATAGCCTCGTT	[25]
		Md-ACO-R	GGTGCTGGGCTGATGAATG	
		Md-ACO	HEX-TACAACCCAGGCAACG	

A +prior to the nucleotide code indicates that the following nucleotide is an LNA

### qPCR setup

Different primer and probe concentrations were tested to determine the optimal working concentration in singleplex and multiplex TaqMan^®^ assays and in SYBR-Green assays. All qPCR reactions were run on a CFX384 Touch™ or CFX96 Touch™ real-time PCR detection system (Bio-Rad) in HardShell^®^ Bio-Rad Plates sealed with Microseal ‘B’ Film (Bio-Rad). PCR evaluation was performed using the CFX Manager software (Bio-Rad).

Sequences for primer and probes that were used as internal standards in this study can be found in Table 2.

Criteria for a high-quality qPCR run with the possibility of the quantification of a target were defined according to the MIQE Guidelines [7].

### SYBR-Green assay

The primer performance was analyzed based on the recommendations of [23]. The optimal annealing temperature was determined by running a qPCR at annealing temperature gradient from 50 to 60 °C. A primer concentration matrix was performed with varying, symmetric and asymmetric primer concentrations, i.e. concentrations ranging from 125 nM to 500 nM. To analyze the effect of MgCl_2_ on the PCR performance, 1.5 mM, 3 mM, 4.5 mM or 6 mM MgCl_2_ were added to the reaction (note: the SYBR FAST qPCR Kit Master Mix already contains 2.5 mM MgCl_2_ in the 1× working concentration and the MgCl_2_ was additionally added to the mix). If not indicated differently, PCR reactions with SYBR-Green were performed as follows: 250 nM UNI28S-fwd, 250 nM UNI28S-rev, 1X SYBR FAST qPCR Kit Master Mix (Kapa Biosystems) with 2 µl template DNA, adjusted to a total volume of 10 µl with nuclease-free water. Cycling conditions were as follows: initial denaturation at 95 °C C for 20 s followed by 35 cycles of 95 °C for 3 s and annealing at 60 °C for 30 s. The generated amplicon was melted from 65 to 95 °C with an increment of 0.5 °C per 5 s.

### Probe-based singleplex assay

The following TaqMan^®^ qPCR mastermix reagent concentrations were adjusted based on the recommendations of [23]. For the amplification of the 28S fragment in a total reaction volume of 10 µl the following components were combined: 2 µl of template DNA, 5 µl 2X iQ™ Multiplex Powermix (Bio-Rad), 400 nM of each primer (UNI28S-fwd, UNI28S-rev) and 200 nM of the probe (UNI28S-P). The primers were synthesized and supplied by Microsynth AG (Switzerland) and the probe, with incorporated LNAs, was supplied by EuroClone S.P.A. (Italy). The following cycling conditions were applied: initial denaturation at 95 °C for 3 min followed by 35 cycles of 95 °C for 15 s and 60 °C for 1 min.

As an example for other internal controls the apple specific chloroplast DNA gene tRNA leucine (*trnL*) [24] and the *Malus* × *domestica* single-copy gene 1-aminocyclopropane-1-carboxylate oxidase (*ACO*) [25] were amplified in a 10 µl reaction volume using 5 µl 2X iQ™ Multiplex Powermix (Bio-Rad), 200 nM qMd-cpLeu-F and qMd-cpLeu-R primer [24] or qMd-ACO-F/qMd-ACO-R primer pair [25] together with 200 nM of the respective probe qMd-cpLeu or qMd-ACO. Both probes are conjugated at the 5′- end to HEX reporter dye. Cycling conditions were the same as for 28S probe-based assay.

### Probe-based multiplex assay

The multiplex qPCR was run in a total reaction volume of 10 µl with 2 µl of sample DNA and 5 µl 2X iQ™ Multiplex Powermix, 900 nM of each SAD-fwd (5′-TGGTTAGAGCACACGCCTGAT-3′) and SAD-rev (5′-TCCACTGTGCGCCCTTAATT-3′) primer, 200 nM of qAP-IGS probe (5′FAM- CAAAGTATTTATCTTAAGAAAACAAGCT-3′) and 200 nM of qPD-IGS probe (5′TexRed- AATATTTATTTTAAAAAAAAGCTCTTTG-3′) [42, 43] together with 400 nM of each UNI28S-fwd and UNI28S-rev and 200 nM of UNI28S-P. PCR conditions were the same as described for the 28S probe-based singleplex assay.

### Plasmid standards for quantification

The amplified 28S rDNA fragment was subcloned into the plasmid vector pJET1.2 via the CloneJET PCR Cloning Kit (ThermoFisher) and transformed into electrocompetent MegaX DH10B T1^R^*E. coli* (Invitrogen). The plasmid was extracted from *E. coli* with the QIAprep Spin Miniprep Kit (Qiagen) according to manufacturer’s instructions. Additionally, to remove genomic DNA impurities, that might affect accurate photometric plasmid quantification, the eluates were run on an agarose gel and the plasmid band was gel extracted (QIAquick Gel Extraction Kit, Qiagen). The plasmid DNA concentration was measured with PicoGreen^®^ (ThermoFisher) on a NanoDrop™ 3300 fluorospectrometer (ThermoFisher). Plasmids were diluted to a concentration of 2.5 × 10^8^ plasmid copies µl^−1^. Plasmid copy content was calculated based on the molecular weight of the plasmid, applying the following formula, considering that one nucleotide has a molecular weight of 327 g/mol:

$${\text{plasmid~copy~content}}~\left[ {\frac{{{\text{copies}}}}{{{\upmu }{\text{l}}}}} \right] = \frac{{{\text{plasmid~concentration}}\left[ {\frac{{{\text{ng}}}}{{{\upmu }{\text{l}}}}} \right]}}{{{\text{molecular~weight~of~the~plasmid~}}\left[ {\frac{{{\text{ng}}}}{{{\text{copy}}}}} \right]}}$$

With the adjusted plasmid solution, a tenfold standard dilution series in AE buffer (Qiagen) for quantification of the 28S rDNA fragment was prepared. The dilution series of 2.5 × 10^6^–2.5 × 10^0^ 28S copies µl^−1^ was analyzed together with each qPCR run to determine qPCR performance parameters such as the linear dynamic range (LDR), amplification efficiency, coefficient of determination (correlation coefficient, R^2^) and the limit of detection (LOD). As described for the 28S fragment, phytoplasmal target sequences of ‘*Candidatus* Phytoplasma mali’ (apple proliferation phytoplasma; AP) and ‘*Candidatus* Phytoplasma pyri’ (pear decline phytoplasma; PD) were subcloned into pJET1.2 plasmid vector and prepared from *E. coli* strain DH10B T1^R^. The subcloned amplicons were amplified with SAD-fwd and SAD-rev primer pair, according to [42] using DNA from infected apple or pear as template. A tenfold dilution series of the three template plasmids (pJET1.2-28S, pJET1.2-AP and pJET1.2-PD) in the range of 2.5 × 10^7^–2.5 × 10^0^ plasmid copies µl^−1^ (of each plasmid) was prepared in AE-buffer (Qiagen). The standard dilution series was used to analyze the performance of every multiplex qPCR run.

### DNA, RNA extraction and cDNA synthesis

To test the specificity of the primer pair and probe, samples from 43 different eukaryotic species were tested. DNA from horse hairs, beef, pork, mutton, roe venison, salmon filet, tick, leafhopper and psyllid species, yeast and bacteria (as a negative control) was extracted using the DNeasy Blood and Tissue Kit (Qiagen) and DNA from plant and fungi species was extracted with the DNeasy Plant Mini Kit (Qiagen), according to the manufacturer’s instructions. DNA was eluted in 100 µL AE buffer provided with the extraction kits. DNA quantity was measured with a NanoDropTM 1000 spectrophotometer. RNA from apple and grapevine was extracted as described in 44 [44]. The extracted RNA was pretreated with TURBO DNA-free™ Kit (Invitrogen). cDNA was synthesized using the SuperScript™ VILO™ cDNA Synthesis Kit (Invitrogen). For comparability of 28S copy numbers DNA was diluted to a final concentration of 5.0 ng µl^−1^ prior use as template in the qPCR assay.

### Statistical analysis

All statistical analyses were performed using GraphPad Prism^®^ 7.01.

## Supplementary information

**Additional file 1.**Additional figures and tables.

## Data Availability

The datasets used and/or analysed during the current study are available from the corresponding author on reasonable request.

## Abbreviations

AP: Apple proliferation
enCtrl: Endogenous control
exCtrl: Exogenous control
LDR: Linear dynamic range
LNA: Locked nucleic acids
LOD: Limit of detection
PD: Pear decline

## References

[1] Kleppe K, Ohtsuka E, Kleppe R, Molineux I, Khorana HG (1971). Studies on polynucleotides: XCVI Repair replications of short synthetic DNA’s as catalyzed by DNA polymerases. J Mol Biol.

[2] Mullis KB, Faloona FA (1987). Specific synthesis of DNA in vitro via a polymerase-catalyzed chain reaction. Methods Enzymol.

[3] Saunders NA, Logan J, Logan JMJ, Edwards KJ, Saunders NA (2009). An introduction to real-time PCR. Real-time PCR: current technology and applications.

[4] Saiki RK, Gelfand DH, Stoffel S, Scharf SJ, Higuchi R, Horn GT (1988). Primer-directed enzymatic amplification of DNA with a thermostable DNA polymerase. Science.

[5] Rezadoost MH, Kordrostami M, Kumleh HH (2016). An efficient protocol for isolation of inhibitor-free nucleic acids even from recalcitrant plants. 3 Biotech..

[6] Hoorfar J, Malorny B, Abdulmawjood A, Cook N, Wagner M, Fach P (2004). Practical considerations in design of internal amplification controls for diagnostic PCR assays. J Clin Microbiol.

[7] Bustin SA, Benes V, Garson JA, Hellemans J, Huggett J, Kubista M (2009). The MIQE guidelines: minimum information for publication of quantitative real-time pcr experiments. Clin Chem.

[8] Hodgetts J, Boonham N, Mumford R, Dickinson M (2009). Panel of 23S rRNA gene-based real-time PCR assays for improved universal and group-specific detection of phytoplasmas. Appl Environ Microbiol.

[9] Kavanagh I, Jones G, Nayab S, Kennedy S, Oswald N (2011). Significance of controls and standard curves in PCR. PCR troubleshooting and optimization: the essential guide.

[10] Christensen NM, Nyskjold H, Nicolaisen M (2013). Real-time PCR for universal phytoplasma detection and quantification. Methods Mol Biol.

[11] Bertaccini A, Duduk B, Paltrinieri S, Contaldo N (2014). Phytoplasmas and phytoplasma diseases: a severe threat to agriculture. Am J Plant Sci..

[12] Maejima K, Oshima K, Namba S (2014). Exploring the phytoplasmas, plant pathogenic bacteria. J Gen Plant Pathol.

[13] Abou-Jawdah Y, Aknadibossian V, Jawhari M, Tawidian P, Abrahamian P, Musetti R, Pagliari L (2019). Real-time PCR protocol for phytoplasma detection and quantification. Phytoplasmas: methods and protocols.

[14] Linck H, Krüger E, Reineke A (2017). A multiplex TaqMan qPCR assay for sensitive and rapid detection of phytoplasmas infecting *Rubus* species. PLoS ONE.

[15] Baric S (2012). Molecular tools applied to the advancement of fruit growing in south tyrol: a review. Erwerbs-Obstbau..

[16] Monti M, Martini M, Tedeschi R (2013). EvaGreen Real-time PCR protocol for specific ‘*Candidatus* Phytoplasma mali’ detection and quantification in insects. Mol Cell Probes.

[17] Pelletier C, Salar P, Gillet J, Cloquemin G, Very P, Foissac X, Malembic-Maher S (2009). Triplex real-time PCR assay for sensitive and simultaneous detection of grapevine phytoplasmas of the 16SrV and 16SrXII-A group with an endogenous analytical control. Vitis..

[18] Ikten C, Ustun R, Catal M, Yol E, Uzun B (2016). Multiplex real-time qPCR assay for simultaneous and sensitive detection of phytoplasmas in sesame plants and insect vectors. PLoS ONE.

[19] Ito T, Suzaki K (2017). Universal detection of phytoplasmas and *Xylella* spp. by TaqMan singleplex and multiplex real-time PCR with dual priming oligonucleotides. PLoS ONE..

[20] Mittelberger C, Obkircher L, Oettl S, Oppedisano T, Pedrazzoli F, Panassiti B (2017). The insect vector *Cacopsylla picta* vertically transmits the bacterium ‘*Candidatus* Phytoplasma mali’ to its progeny. Plant Pathol.

[21] Kaur H, Arora A, Wengel J, Maiti S (2006). Thermodynamic, counterion, and hydration effects for the incorporation of locked nucleic acid nucleotides into DNA duplexes. Biochemistry.

[22] You Y, Moreira BG, Behlke MA, Owczarzy R (2006). Design of LNA probes that improve mismatch discrimination. Nucleic Acids Res.

[23] Bustin S, Huggett J (2017). qPCR primer design revisited. Biomol Detect Quantif..

[24] Baric S, Dalla-Via J (2004). A new approach to apple proliferation detection: a highly sensitive real-time PCR assay. J Microbiol Methods.

[25] Baric S, Berger J, Cainelli C, Kerschbamer C, Letschka T, Dalla-Via J (2011). Seasonal colonisation of apple trees by ‘*Candidatus* Phytoplasma mali’ revealed by a new quantitative TaqMan real-time PCR approach. Eur J Plant Pathol.

[26] Christensen NM, Nicolaisen M, Hansen M, Schulz A (2004). Distribution of phytoplasmas in infected plants as revealed by real-time PCR and bioimaging. Mol Plant Microbe Interact.

[27] Piorkowski G, Baronti C, de Lamballerie X, de Fabritus L, Bichaud L, Pastorino BA, Bessaud M (2014). Development of generic Taqman PCR and RT-PCR assays for the detection of DNA and mRNA of β-actin-encoding sequences in a wide range of animal species. J Virol Methods.

[28] Kozera B, Rapacz M (2013). Reference genes in real-time PCR. J Appl Genet..

[29] Zhong H, Simons JW (1999). Direct comparison of GAPDH, *β*-actin, cyclophilin, and 28S rRNA as Internal standards for quantifying RNA levels under hypoxia. Biochem Biophys Res Commun.

[30] Xue J-L, Salem TZ, Turney CM, Cheng X-W (2010). Strategy of the use of 28S rRNA as a housekeeping gene in real-time quantitative PCR analysis of gene transcription in insect cells infected by viruses. J Virol Methods.

[31] Tsotetsi TN, Collins NE, Oosthuizen MC, Sibeko-Matjila KP (2018). Selection and evaluation of housekeeping genes as endogenous controls for quantification of mRNA transcripts in *Theileria parva* using quantitative real-time polymerase chain reaction (qPCR). PLoS ONE.

[32] Singh S, Gupta M, Pandher S, Kaur G, Rathore P, Palli SR (2018). Selection of housekeeping genes and demonstration of RNAi in cotton leafhopper, *Amrasca biguttula biguttula* (Ishida). PLoS ONE.

[33] Yang X, Pan H, Yuan L, Zhou X (2018). Reference gene selection for RT-qPCR analysis in *Harmonia axyridis*, a global invasive lady beetle. Sci Rep..

[34] Taylor SC, Nadeau K, Abbasi M, Lachance C, Nguyen M, Fenrich J (2019). The ultimate qPCR experiment: producing publication quality, reproducible data the first time. Trends Biotechnol.

[35] D’haene B, Hellemans J. The importance of quality control during qPCR data analysis. Int Drug Discov. 2010:18–31.

[36] Johnson G, Nolan T, Bustin SA, Wilks M (2013). Real-time quantitative PCR, pathogen detection and MIQE. PCR detection of microbial pathogens.

[37] Raymaekers M, Smets R, Maes B, Cartuyvels R (2009). Checklist for optimization and validation of real-time PCR assays. J Clin Lab Anal.

[38] Pfaffl MW, Filion M (2012). Quantification strategies in real time polymerase chain reaction. Quantitative real-time PCR in applied microbiology.

[39] Tajadini M, Panjehpour M, Javanmard SH (2014). Comparison of SYBR Green and TaqMan methods in quantitative real-time polymerase chain reaction analysis of four adenosine receptor subtypes. Adv Biomed Res..

[40] Demeuse KL, Grode AS, Szendrei Z (2016). Comparing qPCR and nested PCR diagnostic methods for aster yellows phytoplasma in Aster Leafhoppers. Plant Dis.

[41] Dietrich CH, Rakitov RA, Holmes JL, Black WC (2001). Phylogeny of the major lineages of Membracoidea (Insecta: Hemiptera: Cicadomorpha) based on 28S rDNA sequences. Mol Phylogenet Evol.

[42] Nikolić P, Mehle N, Gruden K, Ravnikar M, Dermastia M (2010). A panel of real-time PCR assays for specific detection of three phytoplasmas from the apple proliferation group. Mol Cell Probes.

[43] Mehle N, Nikolić P, Gruden K, Ravnikar M, Dermastia M (2013). Real-time PCR for specific detection of three phytoplasmas from the apple proliferation group. Methods Mol Biol.

[44] MacKenzie DJ, McLean MA, Mukerji S, Green M (1997). Improved RNA extraction from woody plants for the detection of viral pathogens by reverse transcription-polymerase chain reaction. Plant Dis.

