# Supplementary material for: Development of a universal endogenous qPCR control for eukaryotic DNA samples
Source: Plant Methods. 2020 Apr 16;16:53. doi: 10.1186/s13007-020-00597-2 (PMC7160944; doi:10.1186/s13007-020-00597-2)
Supplement: Supplementary file 1 — Additional file 1.Additional figures and tables. [file 13007_2020_597_MOESM1_ESM.pdf]

Additional file 1

Table S1 Checklist of MIQE guidelines.

**Experimental design of 28S rDNA (28S) quantitative real-time PCR based on the MIQE-guidelines (Bustin et al. 2009)**	
Definition of experimental and control groups	see Methods section
Number within each group	see Methods section
Assay carried out by the core or investigator's laboratory?	Investigator's laboratory
Acknowledgment of authors' contributions	See Author Contributions section
**Sample**	
Description	see Methods section, 43 different eukaryotic species
Volume/mass of sample processed	see Table 2
Microdissection or macrodissection	Not applicable
Processing procedure	see Methods section
If frozen, how and how quickly?	Insects were frozen immediately after sampling at -80 °C, all other tissues were immediately processed with the respective DNA extraction kit. see Methods section)
If fixed, with what and how quickly?	Not fixed
Sample storage conditions and duration	DNA samples and plasmid standard dilution series were stored at -20°C. RNA samples were stored at -80°C.
**Nucleic acid extraction**	
Procedure and/or instrumentation	see Methods section
Name of kit and details of any modifications	DNeasy Blood and Tissue extraction Kit (Qiagen) or Nucleo Spin® Tissue kit; RNeasy Plant Mini Kit (Qiagen)
Source of additional reagents used	Not applicable
Details of DNase or RNase treatment	RNase used as described in the respective manual for DNA extraction. DNase treatment of RNA with TURBO DNA-free™ Kit (ThermoFisher), prior to cDNA synthesis
Contamination assessment	see Methods section
Nucleic acid quantification	see Methods section
Instrument and method	NanoDrop™ 1000 or NanoDrop™ 3300

Purity (A260/A280)	Not performed
Yield	Not performed
RNA integrity: method/instrument	Not perfromed
RIN/RQI or Cq of 3' and 5' transcripts	Not performed
Electrophoresis traces	Not performed
Inhibition testing	Dilution series of samples; the assay amplifies a target of eukaryotic host DNA as a proxy for PCR-competency of the sample and to quantify host DNA.
Reverse transcription	SuperScript™ VILO™ cDNA Synthesis Kit (ThermoFisher)
**qPCR target information**	
Gene symbol	28S
Sequence accession number	Basis for primer design were different 28S rDNA sequences from 14 Auchenoryncha species
Location of amplicon	cds
Amplicon length	84 bp
In silico specificity screen (BLAST, and so on)	Specificity of the primers and sequence similarity of the amplicon was confirmed using Geneious 11.1.4 sequence assembly.
Pseudogenes, retropseudogenes, or other homologs?	Not applicable
Sequence alignment	See in silico specificity screen
Secondary structure analysis of amplicon	Not analyzed
Location of each primer by exon or intron	Exon
What splice variants are targeted?	Not analyzed
qPCR oligonucleotides	
Primer sequences	UNI28S-fwd: CTACTATCTAGCGAAACC<br>UNI28S-rev: AYTAGAGTCAAGCTCAAC
RTPrimerDB identification number	Not applicable
Probe sequences	AAA+G+A+AG+A+C+C+C+T
Location and identity of any modifications	Hex labelled 5' end and DAB labelled 3' end. Incorporation of LNAs (indicated with +)
Manufacturer of oligonucleotides	Microsynth AG (Balgach, Switzerland), probe was manufactured by EuroClone S.P.A. (Italy)
Purification method	HPLC purified

**qPCR protocol**	
Complete reaction conditions	see Methods section
Reaction volume and amount of DNA	2 µl of DNA in 10 µl total reaction volume
Primer concentrations	400 nM in probe-based assay or 250 nM in SYBR-Green assay
Polymerase identity and concentration	Polymerase included in 2x iQ™ Multiplex Powermix (Biorad) or 2 x SYBR® FAST qPCR Kit Master Mix (Kapa Biosystems)
Kit identity and manufacturer	iQ™ Multiplex Powermix (Biorad)<br>KAPA SYBR® FAST qPCR Master Mix (2X) Kit (Kapa Biosystems)
Exact chemical composition of the buffer	Not applicable
Additives	No additives and no ROX
Manufacturer of plates and catalog number	Hard-Shell® 384-Well PCR Plates (Bio-Rad), catalog number: HSP3801
Complete thermocycling parameters	see Methods section
Reaction setup	Mastermix and template distribution into 384-well plates was performed by hand pipetting.
Manufacturer of qPCR instrument	CFX384 Touch™ Real-Time PCR Detection System, Bio-Rad Laboratories
qPCR validation	
Evidence of optimization	see Methods section
Specificity	see Results section
Cq of NTC	C_q values varied from run to run, but never showed specific product amplification curves.
Calibration curves with slope and y intercept	Slope was between -3.449 (95% PCR efficiency) and -3.207 (105% PCR efficiency).
PCR efficiency calculated from slope	Only runs with a PCR efficiency between 95% and 105% were considered for evaluation.
Confidence intervalls (CI) for PCR efficiency or standard error (SE)	$R^2 \geq 0.99$ as a function of CI and SE
r^2 of calibration curve	≥ 0.99
Linear dynamic range	50-500,000 28S gene copies reaction^{-1}
C_q variation at limit of detection	Not performed
Confidence intervalls throughout range	Not applicable
Evidence for limit of detection	only results within the linear dynamic range were considered for quantification, however LOD could be defined as 5 28S copies reaction^{-1}.

**Data analysis**	
qPCR analysis software	CFX manager™ software (Bio-Rad Laboratories)
Method of C_q determination	Threshold was set at optimum PCR efficiency (nearest 100%).
Outlier identification and disposition	When using SYBR-Green based qPCR melting curves of all samples were analysed
Results for NTCs	NTCs had no specific amplicon melting peak
Justification of number and choice of reference genes	No gene expression study was performed with these primers
Description of normalization method	see Methods section
Number and concordance of biological replicates	Not applicable
Number of technical replicates	All samples were analysed in triplicates
Repeatability (intraassay variation)	Intraassay variation was determined for five independent runs using four different template concentrations (see Fig. 4). The average C_q-value variation of technical tripilcates was maximum 5.1% (based on the average C_q-value) within the same run (see Fig. 4).
Reproducibility (interassay variation)	Based on the comparison of five independent runs with the same four-point standard curve in triplicates and a similar intercept (38.74±0.35) the average C_q-value of each individual standard dilution did not vary more than 3.7% (based on the average C_q-value of all five independent runs) between the assays (see Fig. 4).
Power analysis	Not performed
Statistical methods for results significance	Two-way ANOVA with Tukey's multiple comparison test
Software	GraphPad Prism 7.01
C_q or raw data submission with RDML	See Data Availability Statement section

Details on qPCR performance of endogenous control 28S rDNA assay, determined according to the MIQE guidelines.

Table S2 Annealing temperature gradient

Tm [°C]	Target	Sample	C_q	Mean C_q	Standard Deviation
60.0	28S	DNA from insect (1:10)	16.14	16.43	0.25
	28S	DNA from insect (1:10)	16.52		
	28S	DNA from insect (1:10)	16.62		
59.1	28S	DNA from insect (1:10)	16.58	16.30	0.24
	28S	DNA from insect (1:10)	16.20		
	28S	DNA from insect (1:10)	16.13		
57.6	28S	DNA from insect (1:10)	15.90	15.85	0.06
	28S	DNA from insect (1:10)	15.88		
	28S	DNA from insect (1:10)	15.78		
55.6	28S	DNA from insect (1:10)	15.49	15.45	0.18
	28S	DNA from insect (1:10)	15.61		
	28S	DNA from insect (1:10)	15.26		
54.4	28S	DNA from insect (1:10)	15.73	15.83	0.10
	28S	DNA from insect (1:10)	15.84		
	28S	DNA from insect (1:10)	15.93		
53.4	28S	DNA from insect (1:10)	15.81	15.83	0.10
	28S	DNA from insect (1:10)	15.74		
	28S	DNA from insect (1:10)	15.93		
51.8	28S	DNA from insect (1:10)	16.56	15.94	0.58
	28S	DNA from insect (1:10)	15.83		
	28S	DNA from insect (1:10)	15.42		
50.6	28S	DNA from insect (1:10)	15.50	15.45	0.07
	28S	DNA from insect (1:10)	15.49		
	28S	DNA from insect (1:10)	15.37		
50.0	28S	DNA from insect (1:10)	15.73	15.67	0.08
	28S	DNA from insect (1:10)	15.70		
	28S	DNA from insect (1:10)	15.58		
50.2	28S	NTC* (H_2O)	24.02	24.02	0.05
	28S	NTC* (H_2O)	24.06		
	28S	NTC* (H_2O)	23.97		

In a total reaction volume of 10 µl 500 nM UNI28S-fwd, 500 nM UNI28-rev Primer and 1X SYBR® FAST qPCR Kit Master Mix (Kapa Biosystems) were used.
*the NTCs showed an amplification curve. This is caused by primer dimers in the absence of a template. Adaption of primer concentrations abolished this problem (see Supplementary Table 3)

Fig. S1 Annealing temperature gradient

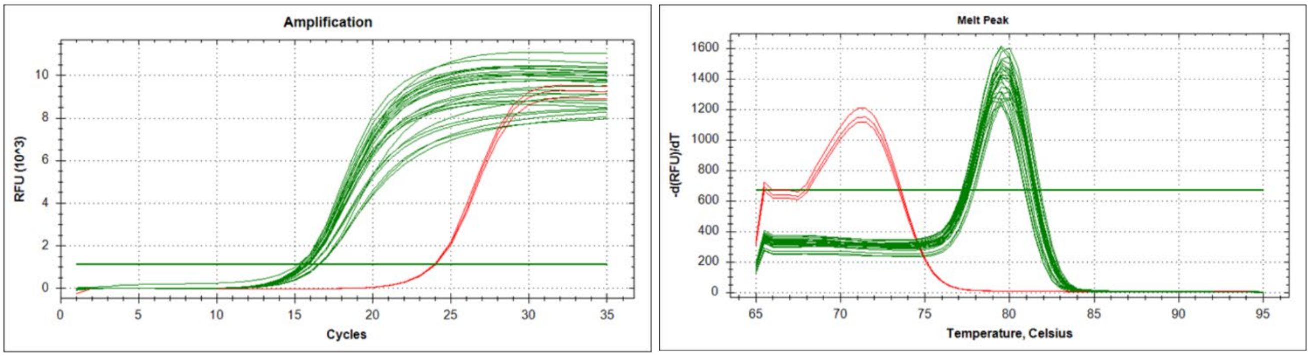

Amplification curves and melt peak of annealing temperature gradient. red line: NTC control (nuclease free water); green line: pool of different insects (1:10 diluted in nuclease free water)

Table S3 Primer matrix

forward primer UNI28-fwd [nM]	reverse primer UNI28-rev [nM]	Target	Sample	C_q	Mean C_q	Standard Deviation
500	500	28S	DNA from insect (1:10)	16.2	16.06	0.11
500	500	28S	DNA from insect (1:10)	16.1		
500	500	28S	DNA from insect (1:10)	15.9		
500	500	28S	NTC (H_2O)	30.9	31.68	1.10
500	500	28S	NTC (H_2O)	32.5		
500	500	28S	NTC (H_2O)	N/A		
500	250	28S	DNA from insect (1:10)	16.9	16.61	0.27
500	250	28S	DNA from insect (1:10)	16.5		
500	250	28S	DNA from insect (1:10)	16.4		
500	250	28S	NTC (H_2O)	N/A	34.46	0.50
500	250	28S	NTC (H_2O)	34.8		
500	250	28S	NTC (H_2O)	34.1		
500	125	28S	DNA from insect (1:10)	27.3	21.04	5.45
500	125	28S	DNA from insect (1:10)	18		
500	125	28S	DNA from insect (1:10)	17.8		
500	125	28S	NTC (H_2O)	N/A	N/A	N/A
500	125	28S	NTC (H_2O)	N/A		
500	125	28S	NTC (H_2O)	N/A		
250	500	28S	DNA from insect (1:10)	17.2	16.83	0.35
250	500	28S	DNA from insect (1:10)	16.7		
250	500	28S	DNA from insect (1:10)	16.6		

250	500	28S	NTC (H_2O)	33	32.75	0.71
250	500	28S	NTC (H_2O)	33.3		
250	500	28S	NTC (H_2O)	32		
250	250	28S	DNA from insect (1:10)	16.8	16.81	0.17
250	250	28S	DNA from insect (1:10)	16.7		
250	250	28S	DNA from insect (1:10)	17		
250	250	28S	NTC (H_2O)	34.3	34.48	0.22
250	250	28S	NTC (H_2O)	N/A		
250	250	28S	NTC (H_2O)	34.6		
250	250	28S	DNA from insect (1:10)	18.1	18.43	0.32
250	250	28S	DNA from insect (1:10)	18.5		
250	250	28S	DNA from insect (1:10)	18.7		
250	250	28S	NTC (H_2O)	N/A	N/A	N/A
250	250	28S	NTC (H_2O)	N/A		
250	250	28S	NTC (H_2O)	N/A		
125	125	28S	DNA from insect (1:10)	18.2	18.49	0.45
125	125	28S	DNA from insect (1:10)	18.3		
125	125	28S	DNA from insect (1:10)	19		
125	125	28S	NTC (H_2O)	N/A	33.38	0.96
125	125	28S	NTC (H_2O)	34.1		
125	125	28S	NTC (H_2O)	32.7		
125	250	28S	DNA from insect (1:10)	18.5	18.47	0.12
125	250	28S	DNA from insect (1:10)	18.6		
125	250	28S	DNA from insect (1:10)	18.4		
125	250	28S	NTC (H_2O)	N/A		
125	250	28S	NTC (H_2O)	N/A		
125	250	28S	NTC (H_2O)	N/A		
125	125	28S	DNA from insect (1:10)	21	21.94	2.59
125	125	28S	DNA from insect (1:10)	19.9		
125	125	28S	DNA from insect (1:10)	24.9		
125	125	28S	NTC (H_2O)	N/A	N/A	N/A
125	125	28S	NTC (H_2O)	N/A		
125	125	28S	NTC (H_2O)	N/A		

Primer matrix of UNI28S-fwd and UNI28S-rev

Fig. S2 Primer matrix

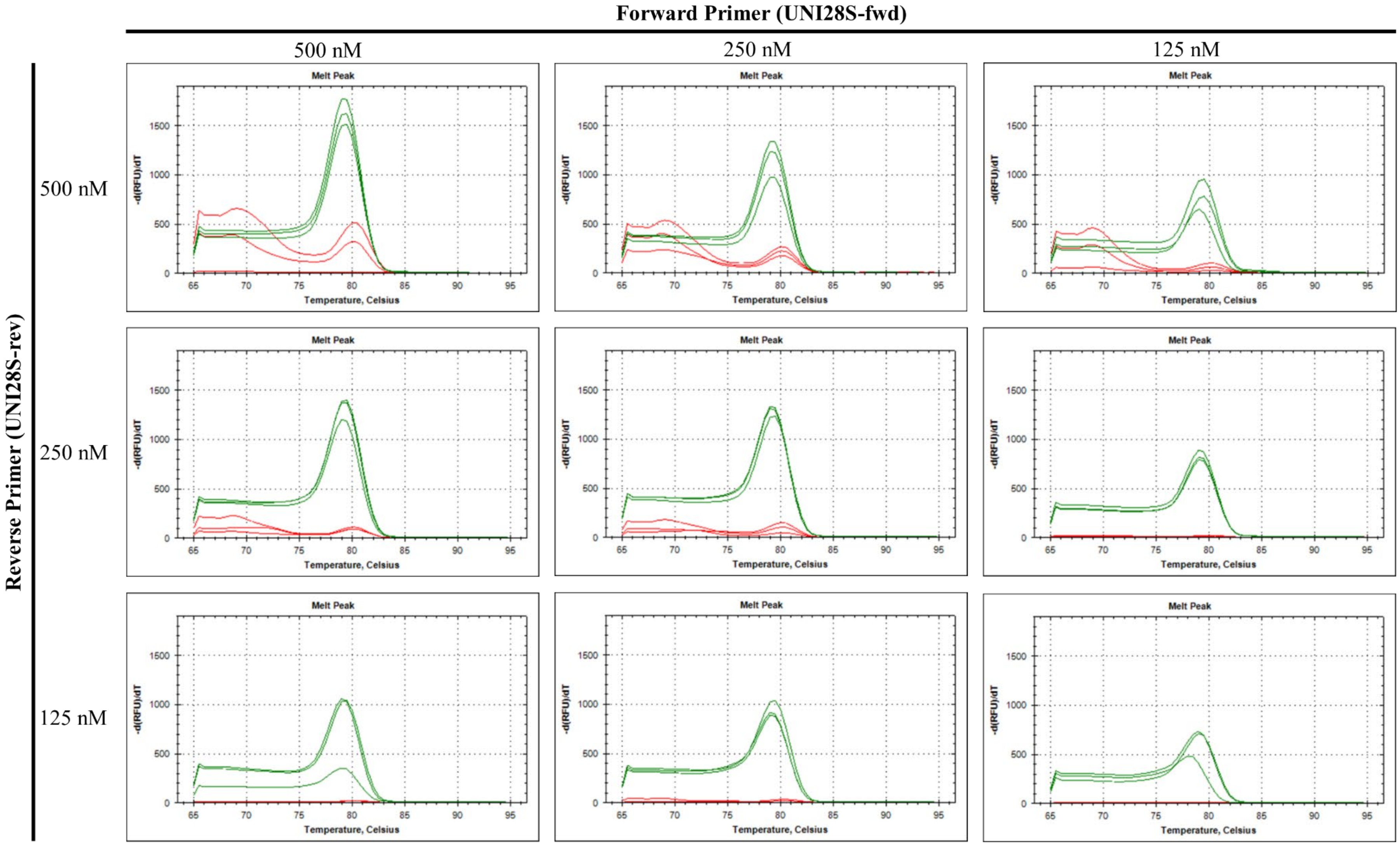

Melt peaks of different forward and reverse primer combinations (primer matrix, see Supplementary Table 3). red line: NTC control (nuclease free water); green line: pool of different insects (1:10 diluted in nuclease free water)

Fig. S3 Effect of $MgCl_2$ concentrations on PCR reaction

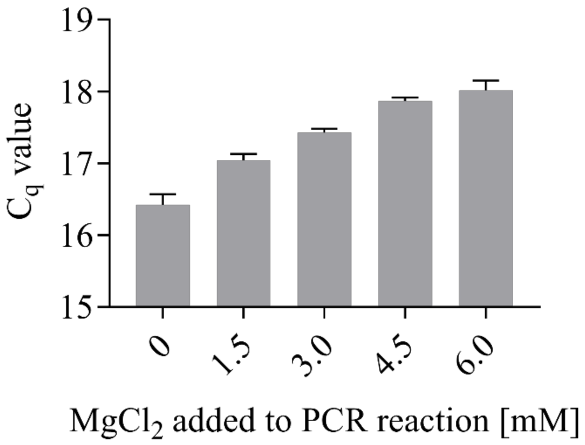

Effect of increased $MgCl_2$ concentrations on 28S amplification with UNI28S-primers.

Table S4 Comparison of different apple specific internal controls

	Sample 1			Sample 2			Sample 3		
	28 S	cpLeu	ACO	28 S	cpLeu	ACO	28 S	cpLeu	ACO
	17.91	15.29	22.49	16.79	15.26	23.16	18.71	15.04	22.74
undiluted	18.28	15.28	22.46	16.99	15.17	22.81	18.69	15.01	22.82
	18.95	15.25	22.19	17.02	15.21	23.07	19.2	14.95	22.75
Mean±SEM	18.38±0.30	15.27±0.01	22.38+0.10	16.93+0.07	15.21+0.03	23.01+0.10	18.87+0.17	15.00+0.03	22.77+0.03
	21.73	18.7	25.78	19.87	18.77	26.43	21.78	18.4	26.41
1:10	21.22	18.81	25.69	20.06	18.04	26.29	21.59	18.46	26.47
	21.2	18.7	25.52	20.64	18.7	26.39	21.51	18.38	26.44
Mean±SEM	21.38±0.17	18.74±0.04	25.66+0.08	20.19+0.23	18.50+0.23	26.37+0.04	21.63+0.08	18.41+0.02	26.44+0.02
	25.18	21.98	29.14	23.5	21.87	29.81	24.95	21.75	29.52
1:100	24.73	21.97	29.21	23.38	21.82	29.67	25.02	21.79	29.66
	24.31	21.97	28.9	23.61	21.99	29.98	25.18	21.73	29.55
Mean±SEM	24.74±0.25	21.97±0.00	29.08+0.09	23.50+0.07	21.89+0.05	29.82+0.09	25.05+0.07	21.76+0.02	29.58+0.04
	28.6	25.47	32.58	27.01	25.37	33.05	28.65	25.38	32.87
1:1,000	28.53	25.57	32.85	26.92	25.45	33.5	28.69	25.36	33.22
	28.37	25.33	32.41	27.14	25.32	32.93	28.37	25.24	33.12
Mean±SEM	28.50±0.07	25.46±0.07	32.61+0.13	27.02+0.06	25.38+0.04	33.16+0.17	28.57+0.10	25.33+0.04	33.07+0.10
	32.77	28.76	35.57	31.14	28.8	N/A	31.93	28.65	N/A
1:10,000	31.68	28.68	N/A	30.79	28.9	N/A	31.99	28.64	N/A
	31.35	28.46	N/A	30.34	28.77	N/A	31.8	28.45	34.81
Mean±SEM	31.93±0.43	28.63±0.09	N/A	30.76+0.23	28.82+0.04	N/A	31.91+0.06	28.58+0.07	N/A

Comparison of the performance of different apple specific internal controls and the 28S amplicon (E=97.2%, R^2=0.99) in a dilution series of three apple root samples. The values represent the C_q values (quantification/threshold cycle) of three technical replicates.
